# Model-based clustering for random hypergraphs

**DOI:** 10.1007/s11634-021-00454-7

**Published:** 2021-06-28

**Authors:** Tin Lok James Ng, Thomas Brendan Murphy

**Affiliations:** 1 School of Computer Science and Statistics, Trinity College Dublin, Dublin, Ireland; 2 School of Mathematics and Statistics, University College Dublin, Dublin, Ireland

**Keywords:** Clustering, Hypergraph, Latent class analysis, Minorization maximization, 62H99, 62P25

## Abstract

A probabilistic model for random hypergraphs is introduced to represent unary, binary and higher order interactions among objects in real-world problems. This model is an extension of the latent class analysis model that introduces two clustering structures for hyperedges and captures variation in the size of hyperedges. An expectation maximization algorithm with minorization maximization steps is developed to perform parameter estimation. Model selection using Bayesian Information Criterion is proposed. The model is applied to simulated data and two real-world data sets where interesting results are obtained.

## Introduction

A large number of random graph models have been proposed (Nowicki and Snijders 2001; Hoff et al. 2002; Handcock et al. 2007; Latouche et al. 2011) to describe complex interactions among objects of interest. Pairwise relationships among objects can be naturally represented as a graph, in which the objects are represented by the vertices, and two vertices are joined by an edge if certain relationship exists between them. While graphs are capable of representing pairwise interactions between objects, they are inadequate to represent unary and higher order interactions that are typically observed in many real-world problems. Examples of data with unary and higher-order interactions include co-authorship on academic papers, co-appearance in movie scenes, and songs performed in a concert.

For example, the study of coauthorship networks of scientists have attracted significant interest in both natural and social sciences (Newman 2001a, b, 2004; Moody 2004; Azondekon et al. 2018). Such networks are typically constructed by connecting two scientists if they have coauthored one or more papers together. However, as we will illustrate below, such representation inevitably results in loss of information while a hypergraph representation naturally preserves all information. A hypergraph is a generalization of a graph in which hyperedges are arbitrary sets of vertices, and can contain any number of vertices. As a result, hypergraphs are capable of representing relationships of any order.

**Fig. 1:**
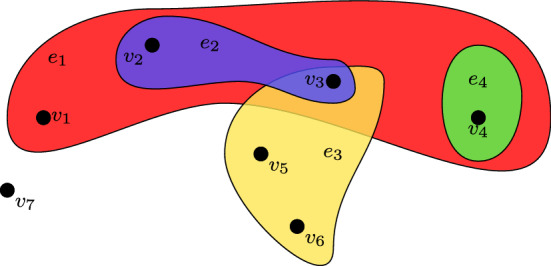
A hypergraph representation of a coauthorship network

We consider a simple example of a coauthorship network with 7 authors and 4 papers, in order to illustrate the benefits of hypergraph modelling. A hypergraph representation of the network is given in Fig. 1, where the vertices $v_1, v_2, \ldots , v_7$ represent the authors while the hyperedges $e_1, \ldots , e_4$ represent the papers. For example, the paper $e_1$ is written by four authors $v_1, v_2$, $v_3$ and $v_4$, the paper $e_2$ is written by two authors $v_2$ and $v_3$, the paper $e_3$ has $v_3$, $v_5$ and $v_6$ as authors and the paper $e_4$ has a single author $v_4$.

On the other hand, a graph representation of this coauthorship network with edges between any two authors who have coauthored at least one paper results in the edge set $\{ (v_1, v_2), (v_1, v_3), (v_1, v_4), (v_2, v_3), (v_2, v_4), (v_3, v_4), (v_3, v_5), (v_3, v_6), (v_5, v_6)\}$. It is evident that much information is lost with this representation. In particular, this representation removes information about the number of authors that co-authored a paper. For example, one can only deduce from this edge set that $v_3$ has co-authored with $v_1$ and $v_2$ while unable to conclude that the co-authorship was for the same paper. Furthermore, the hyperedge $e_4$ which contains a singleton $v_4$ is left out in the graph representation.

A number of random hypergraph models have been studied in probability and combinatorics literature, where theoretical properties are investigated (Karoński and Łuczak 2002; Goldschmidt 2005; de Panafieu 2015; Dyer et al. 2015; Poole 2015). A novel parametrization of distributions on hypergraphs based on the geometry of points is proposed in Lunagómez et al. (2017) which is used to infer Markov structure for multivariate distributions. On the other hand, statistical modeling of random hypergraph data is less developed. Stasi et al. (2014) introduced the hypergraph beta model with three variants, which is a natural extension of the beta model for random graphs (Holland and Leinhardt 1981). In their model, the probability of a hyperedge *e* appearing in the hypergraph is parameterized by a vector $\beta \in \mathbf {R^{N}}$, which represents the “attractiveness” of each vertex. However, their model does not capture clustering among objects, which is a typical real world phenomenon. In addition, the assumption of an upper bound on the size of hyperedges violates the structure of many real world data sets.

**Fig. 2:**
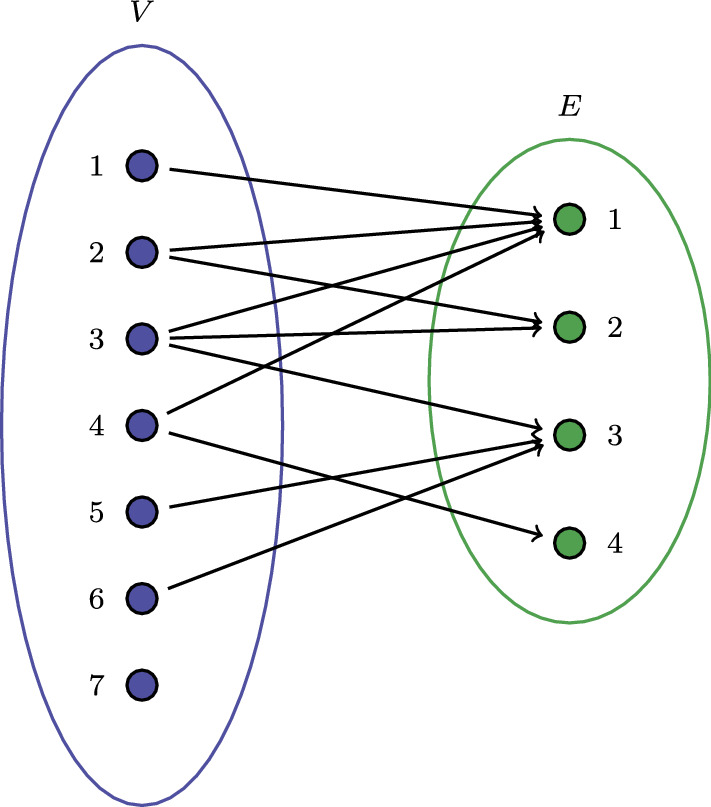
Bipartite graph representation of the hypergraph in Fig. 1

One may equivalently represent a hypergraph using a bipartite network (also called two-mode network and affiliation network). Two-mode networks consist of two different kinds of vertices and edges can only be observed between the two types of vertices, but not between vertices of the same type. A hypergraph can be represented as a two-mode network by considering the hyperedges as a second type of vertices. For example, an equivalent bipartite representation of the hypergraph shown in Fig. 1 is provided in Fig. 2 where the hyperedges $\{ e_1, \ldots , e_4\}$ are now replaced by the four green vertices.

Two-mode networks have been studied in various disciplines including computer science (Perugini et al. 2004), social sciences (Faust et al. 2002; Koskinen and Edling 2012; Friel et al. 2016) and physics (Lind et al. 2005). A number of approaches have been proposed to analyze and model two-mode network data (Borgatti and Everett 1997; Doreian and Batagelj 2004; Latapy et al. 2008; Wang et al. 2009; Snijders et al. 2013; Aitkin et al. 2014). In particular, models originally developed for binary networks were extended for two-mode networks.

Doreian and Batagelj (2004) developes a blockmodeling approach of two-mode network data which aims to simultaneously partition the two types of vertices into blocks. Skvoretz and Faust (1999) proposes the exponential random graph models (ERGMs) for two-mode networks, which models the logit of the probability of an actor belonging to an event as a function of actor and event specific effects and other graph statistics. A clustering algorithm for two-mode networks is developed in Field et al. (2006) based on the modelling framework in Skvoretz and Faust (1999). Several extensions to the ERGMs for bipartite networks are proposed by (eg. Wang et al. 2009, 2013). Snijders et al. (2013) proposes a methodology for studying the co-evolution of two-mode and one-mode networks. A network autocorrelation model for two-mode networks is introduced in Fujimoto et al. (2011). Aitkin et al. (2014) evaluates the identification of clustering structure in bipartite networks through latent class analysis and introduces a new Bayesian method for choosing the number of latent classes.

Representing network observations using two-mode networks has the benefits of modelling vertices of both types jointly. However, in analyzing a two-mode network, one type of vertices may attract most interest. For example, in co-authorship networks, the main interest may lie in the collaborations rather than in co-authored papers. When modeling the co-appearance of characters in the scenes of a movie, one is typically interested in co-appearance of the characters rather than the movie scenes. In such scenarios, a hypergraph representation is most natural by converting one type of vertex into hyperedge.

A related and popular research problem is hypergraph partitioning (Zhou et al. 2007; Leordeanu and Sminchisescu 2012; Purkait et al. 2017). Hypergraph partitioning aims to partition vertices in a hypergraph into clusters based on their higher order interactions, and is an important research problem in computer vision (Agarwal et al. 2006; Li et al. 2013), recommender systems (Bu et al. 2010) and other fields. In contrast, we propose a random hypergraph model which captures the clustering structure of the hyperedges. Since hyperedges are simply arbitrary sets of vertices, interpretable structure within the vertices can also be inferred from the clustering structure of the hyperedges. By adopting a probabilistic approach to hypergraph modeling, the proposed model is capable of quantifying the uncertainties in the clustering of hyperedges.

In this paper, we propose the Extended Latent Class Analysis (ELCA) model for random hypergraphs, which is a natural extension of the Latent Class Analysis (LCA) model (Lazarsfeld and Henry 1968; Goodman 1974; Celeux and Govaert 1991) and includes the LCA model as a special case. The ELCA can alternatively be interpreted as a constrained case of the LCA and it achieves significant reduction in model complexity. Furthermore, the model directly captures the variation in sizes of hyperedges which are typically observed in applications. For example, the number of authors per scientific publication varies widely across different disciplines. We develop an EM (Expectation Maximization) algorithm with MM (Minorization Maximization) steps to perform parameter estimation. To determine the number of latent classes, we employ the Bayesian Information Criterion (BIC). The model is applied to simulated data, and two applications: Star Wars movie scenes and Reuters news articles.

## Model and motivation

### Hypergraph

A hypergraph is represented by a pair $$H = (V,E)$$, where $$V = \{v_{1},v_{2},\ldots ,v_{N}\}$$ is the set of *N* vertices and $$E = \{e_{1}, e_{2}, \ldots , e_{M}\}$$ is the set of *M* hyperedges. A hyperedge *e* is a subset of *V*, and we allow repetitions in the hyperedge set *E*. Thus, the hypergraph *H* can alternatively be represented with a $$ N \times M $$ matrix $$ {\mathbf {X}} = (x_{ij})$$ where $$ x_{ij} = 1 $$ if vertex $$v_{i}$$ appears in hyperedge $$e_{j}$$ and $$ x_{ij}=0$$ otherwise.

### Latent class analysis model for random hypergraphs

The binary latent class analysis (LCA) model (Lazarsfeld and Henry 1968; Goodman 1974) is a commonly used mixture model for high dimensional binary data. It assumes that each observation is a member of one and only one of the *G* latent classes, and conditional on the latent class membership, the manifest variables are mutually independent of each other. The LCA model appears to be a natural candidate to model random hypergraphs where hyperedges are partitioned into *G* latent classes, and the probability that a hyperedge $e \in E$ contains a vertex $v \in V$ depends only on its latent class assignment.

Let ${{\mathbf {X}}} = (x_{ij})$ be the matrix representation of the hypergraph *H* where $x_{ij} = 1$ if vertex $v_i$ is in hyperedge $e_j$ and $x_{ij}=0$ otherwsie. Let $\pi = (\pi _{1}, \ldots , \pi _{G} )$ be the *a priori* latent class membership probabilities, where $\pi _g$ is the probability that a hyperedge belongs to latent class *g*. We define the $N \times G$ matrix ${\mathbf {P}}$, where $p_{ig}$ is the probability that vertex $v_{i}$ is contained in a hyperedge *e* with latent class membership *g*. The probability of observed hyperedge $e_j$, which is represented by $(x_{1j}, \ldots , x_{Nj})$, is thus

$$\begin{aligned} \sum _{g=1}^{G} \pi _{g} \prod _{i=1}^{N} p_{ig}^{x_{ij}} (1-p_{ig})^{1-x_{ij}} . \end{aligned}$$

Thus, the likelihood function of ${\mathbf {P}}$ and $\pi$ can be written as

$$\begin{aligned} L({\mathbf {X}}; {\mathbf {P}}, \pi ) = \prod _{j=1}^{M} \Big [ \sum _{g=1}^{G} \pi _{g} \prod _{i=1}^{N} p_{ig}^{x_{ij}} (1-p_{ig})^{1-x_{ij}} \Big ]. \end{aligned}$$

Let ${\mathbf {Z}}^{(1)}$ be a $M \times G$ latent class membership matrix, where $z^{(1)}_{jg} = 1$ if hyperedge $e_{j}$ has latent class label *g* and $z^{(1)}_{jg}=0$ otherwise. The complete-data likelihood of ${\mathbf {P}}$ and $\pi$ can be expressed as (1).

1 $$\begin{aligned} L({\mathbf {X}}, {\mathbf {Z}}^{(1)}; {\mathbf {P}}, \pi ) = \prod _{j=1}^{M} \prod _{g=1}^{G} \Big [ \pi _{g} \prod _{i=1}^{N} p_{ig}^{x_{ij}} (1-p_{ig})^{1-x_{ij}} \Big ]^{z^{(1)}_{jg}}. \end{aligned}$$

In comparison to the hypergraph beta models introduced in Stasi et al. (2014), the LCA model is capable of capturing the clustering and heterogeneity of hyperedges. For example, academic papers can be naturally labelled according to subject areas and conditional on a paper being labelled mathematics, one would expect that the probability a mathematician co-authored the paper is higher than a biologist. The LCA model does not assume an upper bound on the size of hyperedges and can model hyperedges of any size. Furthermore, an expectation maximization algorithm (Dempster et al. 1977) can be easily derived to perform parameter estimation.

### Extended latent class analysis for random hypergraphs

While the LCA model captures the clustering and heterogeneity of hyperedges in real world data sets, a large number of latent classes are typically required to achieve a good fit of the data. As a result, the number of parameters grows quickly with a moderate or large number of nodes. The complexity of the LCA model can be substantially reduced if we assume that some of the latent class conditional probabilities $(p_{ig})_{i=1}^{N}$ tend to be proportional to each other for different values of *g*. While assuming proportionality of latent class conditional probabilities may appear rather restrictive, it is a reasonable assumption in many hypergraph applications. We develop the Extended Latent Class Analysis (ELCA) model which builds on the proportionality assumption on the conditional probabilities.

Let $a=(a_{1}, \ldots , a_{K})$ with $0 \le a_k \le 1$ be a *K* dimensional vector, the ELCA model assumes that the latent class conditional probabilities are of the form $(\phi _{ig} a_k)_{i=1}^{N}$ for $g=1, \ldots , G$ and $k=1, \ldots , K$. In the context of hypergraph applications, the $a_k$ parameters capture the variations in the size (number of vertices) of the hyperedges whereas the $\phi _{ig}$ values capture the probability that a node is contained in a hyperedge. The ELCA model can be considered as having two types of clustering structure, with the primary clustering structure defined by $\phi _{ig}$ parameters and an additional clustering structure captured by $a_k$ parameters. We note that the ELCA reduces to the standard LCA when $K=1$.

Let $\tau =(\tau _{1},\ldots ,\tau _{K})$ be the clustering assignment probabilities corresponding to the additional structure, the ELCA model assumes that these two clustering structure are *a priori* independent. Thus, the probability that a hyperedge has primary cluster label *g* and additional cluster label *k* is $\pi _g \tau _k$, and the probability that the vertex $v_i$ is contained in a hyperedge from the clusters pair (*g*, *k*) is $a_k \phi _{ig}$, and the probability that the vertex $v_i$ is contained in a hyperedge from the primary cluster *g* is $\phi _{ig} \sum _{k=1}^{K} a_k \tau _k$.

Under the ELCA model with *G* primary clusters and *K* additional clusters, the probability of observing a hyperedge $(x_{1j}, \ldots , x_{Nj})$ is given by

$$\begin{aligned} \sum _{g=1}^{G} \sum _{k=1}^{K} \pi _{g} \tau _{k} \prod _{i=1}^{N} (a_{k} \phi _{ig})^{x_{ij}} (1-a_{k} \phi _{ig})^{1-x_{ij}} . \end{aligned}$$

Let $\theta = (\pi , \tau , \phi , a)$ denote the model parameters, the likelihood function can be written as

$$\begin{aligned} L({\mathbf {X}};\theta ) = \prod _{j=1}^{M} \bigg \{ \sum _{g=1}^{G} \sum _{k=1}^{K} \pi _{g} \tau _{k} \prod _{i=1}^{N} (a_{k} \phi _{ig})^{x_{ij}} (1-a_{k} \phi _{ig})^{1-x_{ij}} \bigg \}. \end{aligned}$$

The ELCA model is not identifiable if the parameters $(a_k)_{k=1}^{K}$ are not constrained. To see this, if $0< a_k < 1$ for all *k*, then the likelihood function is invariant under the transformation $(a_k)_{k=1}^{K} \rightarrow (C a_k)_{k=1}^{K}$ and $(\phi _{ig})_{i=1,g=1}^{i=N,g=G} \rightarrow (C^{-1} \phi _{ig})_{i=1, g=1}^{i=N, g=G}$, where *C* is some positive constant such that $\max _{k} \{C a_k \} \le 1$. Thus, to ensure the identifiability of the model, $(a_k)_{k=1}^{K}$ are ranked by increasing order with $a_K=1$.

We define the $M \times K$ additional cluster membership matrix ${\mathbf {Z}}^{(2)} = (z_{jk}^{(2)})$, where $z_{jk}^{(2)} = 1$ if hyperedge $e_{j}$ has additional cluster label *k* and $z_{jk}^{(2)}=0$ otherwise. The complete data likelihood function of ${\mathbf {X}}$, ${\mathbf {Z}}^{(1)}$ and ${\mathbf {Z}}^{(2)}$ is given as

2 $$\begin{aligned} L({\mathbf {X}},{\mathbf {Z}}^{(1)}, {\mathbf {Z}}^{(2)};\theta ) = \prod _{j=1}^{M} \prod _{g=1}^{G} \prod _{k=1}^{K} \Big [ \pi _{g} \tau _{k} \prod _{i=1}^{N} (a_{k} \phi _{ig})^{x_{ij}} (1-a_{k}\phi _{ig})^{1-x_{ij}} \Big ]^{z^{(1)}_{jg} z^{(2)}_{jk}}. \end{aligned}$$

We note that any ELCA with *G* primary clusters and *K* additional clusters can be equivalently represented as a standard LCA with $G \times K$ clusters. Under the standard LCA representation of the ELCA model, the $G \times K$ vectors of latent class conditional probabilities $\big \{ (p_{ig})_{i=1}^{N} \big \}_{g=1}^{G \times K}$ can be partitioned into *G* sets of equal size *K*, and $(p_{ig})_{i=1}^{N}$ are proportional to each other within each set with the constants of proportionality determined by $(a_k)_{k=1}^{K}$. Consider the ELCA with 2 primary clusters and 2 additional clusters, which is a special case of the 4-cluster LCA model. The probabilities that vertex $v_i$ is contained in a hyperedge from the cluster pair (1, 1), (1, 2), (2, 1), (2, 2) are given by $\phi _{i1}, \phi _{i2}, a_1 \phi _{i1}, a_1 \phi _{i2}$.

It is easy to see that under the proportionality assumption, the ELCA model achieves significant reduction in the number of parameters. For the ELCA model with *G* primary clusters and *K* additional clusters, the number of parameters is given by $$GN + 2(K-1) + (G-1)$$ whereas the number of parameters for the LCA with $$G \times K$$ clusters is $$G K N + (G K - 1) $$.

### Theoretical properties

We analyze the distribution of the size of a random hyperedge under the proposed ELCA model. Proposition 1 below shows that the size of the hyperedges simulated from the ELCA model tend to have larger variance than those simulated from the LCA model.

#### Proposition 1

Suppose we are given the LCA model with parameters $\{ \pi , p \}$ and the ELCA model with parameters $\{ \pi , \tau , a, \phi \}$ and *N* vertices. Suppose the condition $p_{ig} = \phi _{ig} \sum _{k=1}^{K} a_{k} \tau _{k}$ holds for $i=1,\ldots ,N$ and $g=1,\ldots , G$. This condition ensures that the latent class conditional probabilities of the primary clustering structure are the same for both models.

Let *A* denote the cardinality $|e_1|$ of a random hyperedge $e_1$ generated under the LCA model. Similarly, let *B* denote the cardinality $|e_2|$ of a random hyperedge $e_2$ generated under the ELCA model. We have the following results:

$$\begin{aligned} E(A)= & {} E(B) \\ Var(A)\le & {} Var(B) . \end{aligned}$$

#### Proof

The proof is straightforward and is given in the Appendix. $\square$

We now let $f_{N}(y)$ be the probability mass of the size of a random hyperedge simulated from a *G* cluster LCA model. Similarly, we let $h_{N}(y)$ be the probability mass of the size of a random hyperedge simulated from the ELCA model with *G* clusters and *K* additional clusters. The following result can be derived.

#### Proposition 2

1. Under the specifications of a LCA model with parameters $\pi =(\pi _{1},\ldots ,\pi _{G})$ and $\{ p_{ig} \}_{i=1,\ldots ,N, g=1,\ldots ,G}$, and suppose the following conditions hold for $g=1,\ldots ,G$,
  $$\begin{aligned} \lambda _{N}^{(g)}= & {} \sum _{i=1}^{N} p_{ig} \rightarrow \lambda ^{(g)} > 0 \\ \sum _{i=1}^{N} p_{ig}^{2}\rightarrow & {} 0 \end{aligned}$$
  as $N \rightarrow \infty$. We have
  $$\begin{aligned} f_{N}(y) \rightarrow \sum _{g=1}^{G} \pi _{g} \frac{ e^{ -\lambda ^{(g)} } (\lambda ^{(g)})^{y} }{y!} . \end{aligned}$$
  That is, the distribution of the size of a random hyperedge converges to a mixture of Poisson distributions with *G* components.
2. Under the specification of a ELCA model with parameters $\pi =(\pi _{1},\ldots ,\pi _{G})$, $\tau =( \tau _{1}, \ldots , \tau _{K})$, $a=(a_{1}, \ldots , a_{K})$, and $\{ \phi _{ig} \}_{i=1,\ldots ,N, g=1,\ldots ,G}$, and further suppose the following conditions hold for $g=1,\ldots ,G$, and $k=1, \ldots , K$,
  $$\begin{aligned} \lambda _{N}^{(g,k)}= & {} \sum _{i=1}^{N} \phi _{ig} a_{k} \rightarrow \lambda ^{(g,k)} > 0 \\ \sum _{i=1}^{N} \phi _{ig}^{2} a_{k}^{2}\rightarrow & {} 0 \end{aligned}$$
  as $N \rightarrow \infty$. We have
  $$\begin{aligned} h_{N}(y) \rightarrow \sum _{g=1}^{G} \sum _{k=1}^{K} \pi _{g} \tau _{k} \frac{ e^{ - \lambda ^{(g,k)} } ( \lambda ^{(g, k)})^{y} }{y!} . \end{aligned}$$
  That is, the distribution of the size of a random hyperedge converges to a mixture of Poisson distributions with $G \times K$ components.

#### Proof

Conditional on the event that a random hyperedge is generated from cluster *g*, (Wang 1993, Theorem 3) implies that

$$\begin{aligned} f_{N}(y) \rightarrow \frac{e^{-\lambda ^{(g)}} (\lambda ^{(g)})^{y} }{ y! } . \end{aligned}$$

Part 1 result follows by marginalizing over the *G* clusters. The second part of the proposition can be proved similarly. $\square$

Proposition 2 implies that under mild conditions, the distribution of the size of hyperedges converges to a mixture of Poisson distributions with $G \times K$ mixture components as the number of vertices increases. We note that the mixture components of the limiting mixture of Poisson distribution are subject to the same proportionality condition. Nevertheless, larger variations in the size of hyperedges tend to be obtained under the ELCA compared to those obtained under the standard LCA.

## Estimation and model selection

### EM algorithm

We estimate the parameters $\theta = (\pi , \tau , \phi , a)$ of the ELCA model using an EM algorithm (Dempster et al. 1977) which is a popular method in fitting mixture models. The E-step of the EM algorithm involves computing the expected value of the complete data log-likelihood (2) with respect to the distribution of the unobserved ${\mathbf {Z}}^{(1)}$ and ${\mathbf {Z}}^{(2)}$ given the current estimates. The M-step involves maximizing the expected complete data log-likelihood.

Taking logarithm of the complete data likelihood in (2), we obtain the complete data log-likelihood function below.

3 $$\begin{aligned} \log L({\mathbf {X}},{\mathbf {Z}}^{(1)}, {\mathbf {Z}}^{(2)};\theta )= & {} \sum _{j=1}^{M} \sum _{g=1}^{G} \sum _{k=1}^{K} z^{(1)}_{jg} z^{(2)}_{jk} \bigg [ \log \pi _{g} + \log \tau _{k} + \sum _{i=1}^{N} \Big \{ x_{ij} \log (a_{k})\nonumber \\&\quad + \log (\phi _{ig}) + (1-x_{ij}) \log (1-a_{k} \phi _{ig}) \Big \} \bigg ]. \end{aligned}$$

#### E-step

For the E-step, we need to evaluate the expected complete data log-likelihood, which is the expectation of (3) conditional on data *x* and current parameter estimates $\theta ^{(t)}$. The expected complete data log-likelihood is denoted as $Q(\theta |\theta ^{(t)})$ and is defined as

4 $$\begin{aligned} Q(\theta |\theta ^{(t)}) := E(\log L({\mathbf {X}},\mathbf {Z^{(1)}}, \mathbf {Z^{(2)}};\theta )|{\mathbf {X}},\theta ^{(t)}) . \end{aligned}$$

Because the complete-data log-likelihood is linear in $Z_{jg}^{(1)}Z_{jk}^{(2)}$, we need to evaluate the expectation $\widehat{Z^{(1)}_{jg} Z^{(2)}_{jk}} := E(Z^{(1)}_{jg} Z^{(2)}_{jk}|{\mathbf {X}}, \theta ^{(t)})$. We have that

5 $$\begin{aligned} E(Z^{(1)}_{jg} Z^{(2)}_{jk} | {\mathbf {X}}, \theta ^{(t)})= & {} Pr(Z^{(1)}_{jg} = Z^{(2)}_{jk} = 1 | {\mathbf {X}}, \theta ^{(t)}) \nonumber \\= & {} \frac{ \pi ^{(t)}_{g} \tau ^{(t)}_{k} \Big [ \prod _{i=1}^{N} (a_{k} \phi _{ig})^{x_{ij}} (1-a_{k}\phi _{ig})^{1-x_{ij}} \Big ]}{ \sum _{g=1}^{G} \sum _{k=1}^{K} \pi ^{(t)}_{g} \tau ^{(t)}_{k} \Big [ \prod _{i=1}^{N} (a_{k} \phi _{ig})^{x_{ij}} (1-a_{k}\phi _{ig})^{1-x_{ij}} \Big ] }.\qquad \end{aligned}$$

In particular, the E-step has a computational complexity of ${{\mathcal {O}}}(N)$ for each pair (*g*, *k*), and an overall complexity of ${{\mathcal {O}}}(NGK)$.

#### M-step

While the E-step of the EM algorithm is straightforward, the M-step involves complicated maximization. For the M-step, we need to maximize $Q(\theta |\theta ^{(t)})$ with respect to the model parameters $\{ \phi _{ig} \}$, $\{ a_{k} \}$, $\{ \pi _{g} \}$ and $\{ \tau _{k} \}$. Thus, we use the ECM algorithm (Meng and Rubin 1993) which replaces the complex M-step by a series of simpler conditional maximizations. The conditional maximizations with respect to the parameters $\phi$ and *a* do not have closed form solutions. We utilize the MM algorithm (Lange et al. 2000; Hunter and Lange 2004) which works by lower bounding the objective function by a minorizing function and then maximizing the minorizing function. Since the M-step involves a series of conditional maximization, the *Q* function is guaranteed to increase (Meng and Rubin 1993, Theorem 1).

**Maximize w.r.t.** $$\phi _{ig}$$

For fixed *i* and *g*, the objective function retaining terms involving $\phi _{ig}$ can be written as

6 $$\begin{aligned} Q = \sum _{j=1}^{M} \sum _{k=1}^{K} \widehat{Z^{(1)}_{jg} Z^{(2)}_{jk}} \Big ( x_{ij} \log (\phi _{ig}) + (1-x_{ij}) \log (1-a_{k}\phi _{ig}) \Big ) . \end{aligned}$$

An analytic expression for ${{\,\mathrm{arg\,max}\,}}_{\phi _{ig}}\{Q\}$ does not exist due to the $\log (1-a_{k}\phi _{ig})$ term and thus we apply the MM (Minorization Maximization) algorithm (Hunter and Lange 2004). We first apply a quadratic lower bound on the concave function $\log (1-a_{k}\phi _{ig})$ for $k < K$:

$$\begin{aligned} \log (1-a_{k} \phi _{ig})\ge & {} \log (1-a_{k} \phi ^{(t)}_{ig}) + \Big ( \frac{-a_{k}}{1-a_{k}\phi ^{(t)}_{ig}} \Big ) (\phi _{ig}-\phi ^{(t)}_{ig}) \\&+ \frac{1}{2} \Big ( \frac{-a_{k}^{2}}{(1-a_{k})^{2}} \Big ) (\phi _{ig} - \phi ^{(t)}_{ig})^{2} . \end{aligned}$$

Hence, the objective function in (6) up to an additive constant can be minorized by $Q_{lower}$:

7 $$\begin{aligned} Q_{lower}= & {} \sum _{j=1}^{M} \sum _{k=1}^{K} \widehat{Z^{(1)}_{jg} Z^{(2)}_{jk}} x_{ij} \log (\phi _{ig}) + \sum _{j=1}^{M} \sum _{k=1}^{K-1} \widehat{Z^{(1)}_{jg} Z^{(2)}_{jk}} (1-x_{ij}) \nonumber \\&\Bigg ( \Big ( \frac{-a_{k}}{1-a_{k}\phi ^{(t)}_{ig}} \Big ) \phi _{ig} + \frac{1}{2} \Big ( \frac{-a_{k}^{2}}{(1-a_{k})^{2}} \Big ) (\phi _{ig} - \phi ^{(t)}_{ig})^{2} \Bigg ) \nonumber \\&+ \sum _{j=1}^{M} \widehat{Z_{jg}^{(1)} Z_{jK}^{(2)} } (1-x_{ij}) \log (1-\phi _{ig}) . \end{aligned}$$

To simplify the expression above, we define the quantities below:

$$\begin{aligned} A_{1}= & {} \sum _{j=1}^{M} \sum _{k=1}^{K} \widehat{Z^{(1)}_{jg} Z^{(2)}_{jk}} x_{ij} \\ A_{2}= & {} \sum _{j=1}^{M} \widehat{Z^{(1)}_{jg} Z^{(2)}_{jK}} (1-x_{ij}) \\ B_{1}= & {} \sum _{j=1}^{M} \sum _{k=1}^{K-1} \widehat{Z^{(1)}_{jg} Z^{(2)}_{jk}} (1-x_{ij}) \frac{-a_{k}}{1-a_{k} \phi _{ig}^{(t)}} \\ B_{2}= & {} \sum _{j=1}^{M} \sum _{k=1}^{K-1} \widehat{Z^{(1)}_{jg} Z^{(2)}_{jk}} (1-x_{ij}) \frac{1}{2} \frac{-a_{k}^{2}}{(1-a_{k})^{2}} . \end{aligned}$$

Now, the lower bound (7) can be written as below.

$$\begin{aligned} Q_{lower} = A_{1} \log (\phi _{ig}) + A_{2} \log (1-\phi _{ig}) + B_{1} \phi _{ig} + B_{2} (\phi _{ig}-\phi _{ig}^{(t)})^{2} . \end{aligned}$$

Taking derivative with respect to $\phi _{ig}$, we have

$$\begin{aligned} \frac{A_{1}}{\phi _{ig}} - \frac{A_{2}}{1-\phi _{ig}} + B_{1} + 2B_{2}\phi _{ig} -2B_{2}\phi _{ig}^{(t)} = 0 . \end{aligned}$$

Let $C=B_{1} - 2B_{2} \phi ^{(t)}_{ig}$, we have

8 $$\begin{aligned} \phi _{ig}^{3} - \frac{2B_{2}-C}{2B_{2}} \phi _{ig}^{2} - \frac{C-A_{1}-A_{2}}{2B_{2}} \phi _{ig} - \frac{A_{1}}{2B_{2}} = 0 . \end{aligned}$$

Solving the cubic equation above results in the update for $\phi _{ig}$.

**Maximize w.r.t.** $$a_{k}$$

For a fixed *k*, the objective function (3) retaining terms involving $a_{k}$ can be expressed as

9 $$\begin{aligned} Q = \sum _{j=1}^{M} \sum _{g=1}^{G} \widehat{Z^{(1)}_{jg} Z^{(2)}_{jk}} \Big ( \sum _{i=1}^{N} x_{ij} \log (a_{k}) + (1-x_{ij}) \log (1-a_{k}\phi _{ig}) \Big ) . \end{aligned}$$

Since an analytic expression for ${{\,\mathrm{arg\,max}\,}}_{a_{k}}\{Q\}$ does not exist due to the $\log (1-a_{k}\phi _{ig})$ term, we apply the MM (Minorization Maximization) algorithm. We first apply a quadratic lower bound on the concave function

$$\begin{aligned} \log (1-a_{k} \phi _{ig})\ge & {} \log (1-a^{(t)}_{k} \phi _{ig}) + \Big ( \frac{-\phi _{ig}}{1-a_{k}^{(t)} \phi _{ig}} \Big ) (a_{k}-a_{k}^{(t)})\\&+ \frac{1}{2} \Big ( \frac{-\phi _{ig}^{2}}{(1-\phi _{ig})^{2} }\Big ) (a_{k}-a_{k}^{(t)})^{2} . \end{aligned}$$

Hence, (9) up to an additive constant can be minorized by the function:

10 $$\begin{aligned} Q_{lower}= & {} \Bigg ( \sum _{j=1}^{M} \sum _{g=1}^{G} \widehat{Z_{jg}^{(1)} Z_{jk}^{(2)}} \sum _{i=1}^{N} x_{ij} \Bigg ) \log (a_{k}) + \sum _{j=1}^{M} \sum _{g=1}^{G} \widehat{Z_{jg}^{(1)} Z_{jk}^{(2)}} \sum _{i=1}^{N} (1-x_{ij}) \nonumber \\&\Bigg ( \frac{-\phi _{ig}}{1-a_{k}^{(t)} \phi _{ig}} a_{k} + \frac{1}{2} \Big (\frac{-\phi _{ig}^{2}}{(1-\phi _{ig})^{2}} \Big )(a_{k}-a_{k}^{(t)})^{2} \Bigg ) . \end{aligned}$$

To simply the expression above, we define the following quantities:

$$\begin{aligned} A= & {} \sum _{j=1}^{M} \sum _{g=1}^{G} \widehat{Z_{jg}^{(1)} Z_{jk}^{(2)}} \sum _{i=1}^{N} x_{ij} \\ B= & {} \sum _{j=1}^{M} \sum _{g=1}^{G} \widehat{Z_{jg}^{(1)} Z_{jk}^{(2)}} \sum _{i=1}^{N} (1-x_{ij}) \Big ( \frac{-\phi _{ig}}{1-a_{k}^{(t)} \phi _{ig}} \Big ) \\ C= & {} \sum _{j=1}^{M} \sum _{g=1}^{G} \widehat{Z_{jg}^{(1)} Z_{jk}^{(2)}} \sum _{i=1}^{N} (1-x_{ij}) \frac{1}{2} \Big (\frac{-\phi _{ig}^{2}}{(1-\phi _{ig})^{2}} \Big ) . \end{aligned}$$

Taking derivative of (9) with respect to $a_{k}$, we have

$$\begin{aligned} \frac{ \partial Q_{lower}}{\partial a_{k}} = \frac{A}{a_{k}} + B + 2C(a_{k}-a_{k}^{(t)}) = 0 . \end{aligned}$$

Let $D = (\frac{B}{2C} - a_{k}^{(t)})$, $E=-\frac{A}{2C}$, we have

11 $$\begin{aligned} {\hat{a}}_{k} = \Big ( E+\frac{D^{2}}{4} \Big )^{1/2} - \frac{D}{2} . \end{aligned}$$

**Maximize w.r.t.** $\pi _{g}$ **and** $\tau _{k}$ We apply the method of Lagrange multipliers to derive the updates for $\pi _g$ and $\tau _k$. The objective function for $(\pi _h)_{h=1}^{G}$ is given by

12 $$\begin{aligned} \sum _{j=1}^{M} \sum _{h=1}^{G} \sum _{k=1}^{K} \widehat{ z^{(1)}_{jh} z^{(2)}_{jk} } \log \pi _{h} + \lambda \bigg (1 - \sum _{h=1}^{G} \pi _h \bigg ), \end{aligned}$$

where $\lambda$ is the Lagrange multipler. Differentiating w.r.t. $\pi _g$ and setting to 0 gives

$$\begin{aligned} \frac{ \sum _{j=1}^{M} \sum _{k=1}^{K} \widehat{ z^{(1)}_{jg} z^{(2)}_{jk} } }{ \pi _g } - \lambda = 0 . \end{aligned}$$

Therefore, the update for $\pi _g$ is given by

13 $$\begin{aligned} {\hat{\pi }}_{g} \propto \sum _{j=1}^{M} \sum _{k=1}^{K} \widehat{ Z_{jg}^{(1)} Z_{jk}^{(2)} } . \end{aligned}$$

The update for $\tau _k$ can be derived analogously and is given below:

14 $$\begin{aligned} {\hat{\tau }}_{k} \propto \sum _{j=1}^{M} \sum _{g=1}^{G} \widehat{ Z_{jg}^{(1)} Z_{jk}^{(2)} } . \end{aligned}$$

The EM algorithm is summarized in Algorithm 1, where line 4 corresponds to the expectation step and line 5 - 18 are the conditional maximization steps. In particular, we note that the computational complexity for maximizing $\phi _{ig}$ and $a_{k}$ are given by ${{\mathcal {O}}}(N_{iter}MK)$ and ${{\mathcal {O}}}(N_{iter}MGN)$, respectively, where $N_{iter}$ is the number of iterations required for the MM algorithm.
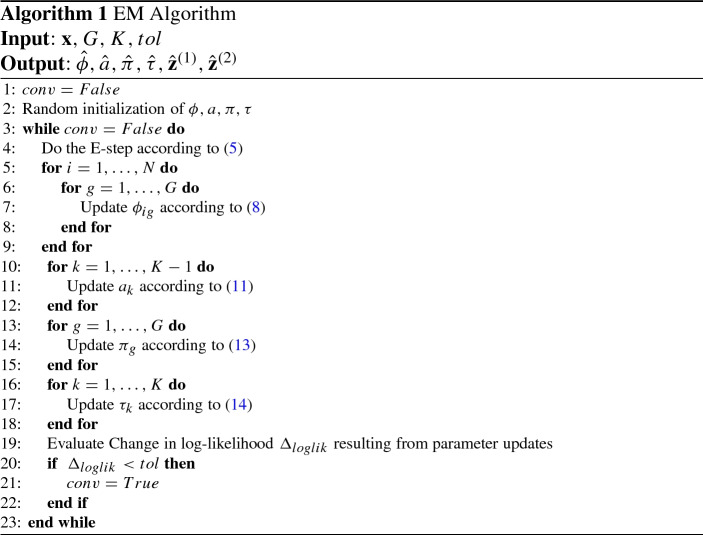


### Model selection

We use the Bayesian Information Criterion (BIC) (Schwarz 1978) to determine the optimal number of primary and additional clusters for the ELCA model. For the ELCA model, the BIC takes the following form:

$$\begin{aligned} -2 \log L + (G N + 2(K-1) + (G-1)) \log M \end{aligned}$$

where $\log L$ is the log-likelihood evaluated at the estimated parameters, and $G N + 2(K-1) + (G-1)$ is the number of parameters in the model. The model with the lowest BIC value is selected. The accuracy of the BIC as a model selection criterion requires *M* to be relatively large compared to *N*. For the standard latent class models, existing literature suggests that the BIC is a good indicator of the true number of classes (Collins et al. 1993) and extensive simulation studies were performed in Nylund et al. (2007) to validate this claim. The performance of BIC as a model selection criterion for the ELCA model is assessed using simulation studies in Sect. 4.

## Simulation studies

We conduct simulation studies to examine the performance of the proposed EM algorithm for the ELCA model and the behavior of BIC as a model selection criterion. The results presented in Tables 1 and 2 are concerned with assessing the convergence behavior of the proposed EM algorithm with various latent class assignment probabilities for primary and additional clusters. Hyperedges are simulated from the ELCA model with two primary clusters and two additional clusters in Table 1 and from the ELCA model with three primary clusters and two additional clusters in Table 2. The specific model parameters used in the simulation are given in the Appendix.

For the model parameters $\phi$, *a*, $\pi$ and $\tau$ of the ELCA model, the $\ell _2$ distances between the true parameters and the estimated ones are presented in Tables 1 and 2 . The misclassification rates for both the primary and additional clusters are also presented. We observe that the estimated parameters converge to the true values as the number of hyperedges increases. It is worth noting that the convergence tends to be faster in the case of two primary clusters compared to three primary clusters.

We examine the performance of BIC in choosing the optimal number of primary and additional clusters. The values in Tables 3 and 4 are computed by comparing the BIC across a range of models, then identifying where the lowest values occurred across these models considered. The model parameters which generate the hyperedges are given in Appendix. For example, with 10 vertices and 200 hyperedges, the lowest values of BIC occurred at the two primary and two additional cluster model (which is the true model) 67% of the time. Looking across the values in Tables 3 and 4, we notice that the BIC tends to be a less accurate model selection criterion when the number of hyperedges is small but improves significantly as the number of hyperedges *M* increases.

As a final simulation study, we simulate hyperedges from the LCA models with two and three clusters and note that they are special cases of the ELCA models with $K=1$ additional cluster. The simulated data is then fitted with the ELCA models with $K=2$ and $K=3$ additional clusters. For various simulation settings, we simulate 100 sets of hyperedges and examine the proportion of times that the true model can be recovered. The true model is considered to be recovered if the estimated parameters satisfy $\max \{\tau _k\} > 1 - \epsilon$ or $\min \{a_k\} > 1 -\epsilon$ for some small positive number $\epsilon$. Simulation results are shown in Table 5 with $\epsilon$ is set to 0.01 and 0.05. We see that using the less strict threshold $\epsilon = 0.05$, the true model is recovered the majority of times across all simulation settings. We also observe that as the number of nodes *N* increases, the proportion of times that the true model is recovered increases considerably. On the other hand, there is no clear relationship between the number of hyperedges *M* and the proportion of successful recovery of the true model.

**Table 1:** Convergence analysis of the EM algorithm for the ELCA model with 2 primary clusters and 2 additional clusters

Model	*M*	$\phi$	*a*	$\pi$	$\tau$	$mis_1$	$mis_2$
10-node ($\pi = (1/2, 1/2)$, $\tau = (1/2,1/2)$)	100	0.0465	0.0224	0.0269	0.0630	0.0412	0.1561
	500	0.0205	0.0075	0.0083	0.0315	0.0374	0.1463
	1000	0.0124	0.0043	0.0064	0.0199	0.0379	0.1428
10-node ($\pi = (2/3, 1/3)$, $\tau = (1/2,1/2)$)	100	0.0549	0.0292	0.0147	0.0491	0.0293	0.1450
	500	0.0248	0.0108	0.0082	0.0296	0.0266	0.1454
	1000	0.0209	0.0046	0.0039	0.0199	0.0273	0.1453
10-node ($\pi = (1/2, 1/2)$, $\tau = (2/3,1/3)$)	100	0.0546	0.0176	0.0173	0.0435	0.0380	0.1332
	500	0.0257	0.0053	0.0106	0.0220	0.0374	0.1328
	1000	0.0146	0.0027	0.0053	0.0173	0.0362	0.1312
10-node ($\pi = (2/3, 1/3)$, $\tau = (2/3,1/3)$)	100	0.0698	0.0137	0.0213	0.0441	0.0365	0.1430
	500	0.0247	0.0082	0.0094	0.0189	0.0372	0.1279
	1000	0.0168	0.0040	0.0082	0.0132	0.0358	0.1235
20-node ($\pi = (1/2, 1/2)$, $\tau = (1/2,1/2)$)	100	0.0559	0.0120	0.0216	0.0195	0.0065	0.0750
	500	0.0170	0.0039	0.0102	0.0103	0.0059	0.0720
	1000	0.0114	0.0037	0.0051	0.0101	0.0056	0.0701
20-node ($\pi = (2/3, 1/3)$, $\tau = (1/2,1/2)$)	100	0.0450	0.0127	0.0250	0.0301	0.0102	0.0640
	500	0.0232	0.0041	0.0080	0.0087	0.0061	0.0620
	1000	0.0112	0.0024	0.0054	0.0082	0.0062	0.0624
20-node ($\pi = (1/2, 1/2)$, $\tau = (2/3,1/3)$)	100	0.0389	0.0120	0.0278	0.0309	0.0090	0.0635
	500	0.0242	0.0040	0.0081	0.0133	0.0089	0.0613
	1000	0.0135	0.0018	0.0077	0.0112	0.0086	0.0604
20-node ($\pi = (2/3, 1/3)$, $\tau = (2/3,1/3)$)	100	0.0558	0.0100	0.0172	0.0304	0.0082	0.0724
	500	0.0194	0.0039	0.0139	0.0121	0.0068	0.0686
	1000	0.0108	0.0021	0.0071	0.0061	0.0067	0.0627

The $\ell _2$ distance between the true parameters of $\phi , a, \pi , \tau$ and the estimated ones, and the misclassification rates for both the primary ($mis_1$) and additional clusters ($mis_2$) are presented

**Table 2:** Convergence analysis of the EM algorithm for the ELCA model with 3 primary clusters and 2 additional clusters

Model	*M*	$\phi$	*a*	$\pi$	$\tau$	$mis_1$	$mis_2$
10-node ($\pi = (1/3, 1/3, 1/3)$, $\tau = (1/2,1/2)$)	100	0.1286	0.0399	0.0235	0.0778	0.1997	0.1858
	500	0.0747	0.0076	0.0108	0.0352	0.1758	0.1692
	1000	0.0541	0.0069	0.0099	0.0138	0.1575	0.1553
10-node ($\pi = (1/2, 1/4, 1/4)$, $\tau = (1/2,1/2)$)	100	0.1317	0.0368	0.0589	0.0590	0.1715	0.1620
	500	0.0850	0.0117	0.0448	0.0363	0.1582	0.1573
	1000	0.0534	0.0052	0.0216	0.0173	0.1529	0.1542
10-node ($\pi = (1/3, 1/3, 1/3)$, $\tau = (3/4,1/4)$)	100	0.1329	0.0432	0.0277	0.0522	0.2335	0.1375
	500	0.1053	0.0106	0.0126	0.0160	0.2172	0.1318
	1000	0.0698	0.0063	0.0112	0.0171	0.2038	0.1291
10-node ($\pi = (1/2, 1/4, 1/4)$, $\tau = (3/4,1/4)$)	100	0.1318	0.0390	0.0782	0.0319	0.2162	0.1485
	500	0.0866	0.0091	0.0521	0.0162	0.1941	0.1292
	1000	0.0745	0.0052	0.0368	0.0158	0.1877	0.1241
20-node ($\pi = (1/3, 1/3, 1/3)$, $\tau = (1/2,1/2)$)	100	0.1083	0.0194	0.0208	0.0390	0.1655	0.1105
	500	0.0523	0.0039	0.0058	0.0139	0.1293	0.1045
	1000	0.0356	0.0019	0.0028	0.0069	0.1208	0.1014
20-node ($\pi = (1/2, 1/4, 1/4)$, $\tau = (1/2,1/2)$)	100	0.1217	0.0169	0.0597	0.0398	0.1647	0.1020
	500	0.0618	0.0062	0.0271	0.0182	0.1176	0.0992
	1000	0.0339	0.0027	0.0139	0.0078	0.1094	0.0967
20-node ($\pi = (1/3, 1/3, 1/3)$, $\tau = (3/4,1/4)$)	100	0.1079	0.0205	0.0290	0.0389	0.2275	0.0915
	500	0.0672	0.0083	0.0104	0.0229	0.1728	0.0862
	1000	0.0434	0.0041	0.0038	0.0131	0.1574	0.0807
20-node ($\pi = (1/2, 1/4, 1/4)$, $\tau = (3/4,1/4)$)	100	0.1265	0.0604	0.0703	0.0389	0.1982	0.0880
	500	0.0724	0.0192	0.0384	0.0207	0.1617	0.0752
	1000	0.0366	0.0025	0.0121	0.0119	0.1426	0.0713

The $\ell _2$ distance between the true parameters of $\phi , a, \pi , \tau$ and the estimated ones, and the misclassification rates for both the primary ($mis_1$) and additional clusters ($mis_2$) are presented

**Table 3:** Percentage of times the lowest BIC values occurred in each model

${\mathbf {G}}$	${\mathbf {K}}$	$N=10$			$N=20$			$N=40$		
		$M = 50$	$M = 200$	$M = 500$	$M = 50$	$M = 200$	$M = 500$	$M = 50$	$M = 200$	$M = 500$
		BIC	BIC	BIC	BIC	BIC	BIC	BIC	BIC	BIC
1	1	0	0	0	0	0	0	0	0	0
1	2	0	0	0	0	0	0	0	0	0
2	1	**52**	28	11	26	14	9	12	0	0
**2**	**2**	42	**67**	**74**	**67**	**82**	**91**	**88**	**100**	**83**
3	1	4	2	8	0	0	0	0	0	0
3	2	2	3	7	7	4	0	0	0	17
4	1	0	0	0	0	0	0	0	0	0
4	2	0	0	0	0	0	0	0	0	0
5	1	0	0	0	0	0	0	0	0	0
5	2	0	0	0	0	0	0	0	0	0

For the first two columns (Column ‘G’ and ‘K’): bold indicates the true model. For the rest of the columns, the largest values are bolded

**Table 4:** Percentage of times the lowest BIC values occurred in each model

${\mathbf {G}}$	${\mathbf {K}}$	$N=10$			$N=20$			$N=40$		
		$M = 50$	$M = 200$	$M = 500$	$M = 50$	$M = 200$	$M = 500$	$M = 50$	$M = 200$	$M = 500$
		BIC	BIC	BIC	BIC	BIC	BIC	BIC	BIC	BIC
1	1	0	0	0	0	0	0	0	0	0
1	2	0	0	0	0	0	0	0	0	0
2	1	**43**	12	2	27	4	0	13	0	0
2	2	28	13	1	**42**	9	0	**58**	2	0
3	1	14	29	19	9	14	7	0	0	0
**3**	**2**	15	**46**	**78**	22	**73**	**84**	29	**98**	**100**
4	1	0	0	0	0	0	6	0	0	0
4	2	0	0	0	0	0	3	0	0	0
5	1	0	0	0	0	0	0	0	0	0
5	2	0	0	0	0	0	0	0	0	0

For the first two columns (Column ‘G’ and ‘K’): bold indicates the true model. For the rest of the columns, the largest values are bolded

**Table 5:** Proportion of times that the true model can be recovered

True model	Fitted model	*N*	*M*	RR ($\epsilon =0.01$)	RR ($\epsilon = 0.05$)
$G=2, K=1$	$G=2, K=2$	10	50	0.55	0.83
			100	0.57	0.83
			500	0.66	0.91
$G=2, K=1$	$G=2, K=2$	20	50	0.64	0.85
			100	0.66	0.83
			500	0.72	0.88
$G=2, K=1$	$G=2, K=3$	10	50	0.32	0.51
			100	0.27	0.59
			500	0.33	0.62
$G=2, K=1$	$G=2, K=3$	20	50	0.56	0.78
			100	0.55	0.86
			500	0.59	0.83
$G=3, K=1$	$G=3, K=2$	10	50	0.55	0.77
			100	0.53	0.80
			500	0.52	0.78
$G=3, K=1$	$G=3, K=2$	20	50	0.70	0.93
			100	0.67	0.92
			500	0.66	0.89
$G=3, K=1$	$G=3, K=3$	10	50	0.43	0.65
			100	0.36	0.63
			500	0.33	0.64
$G=3, K=1$	$G=3, K=3$	20	50	0.54	0.71
			100	0.49	0.76
			500	0.53	0.75

The recovery rates corresponding to $\epsilon =0.01$ and $\epsilon =0.05$ for each simulation setting are shown

## Applications

### Star Wars Movie Scenes

Our first application is modeling co-appearance of the main characters in the scenes of the movie “Star Wars: A New Hope”. We collected the scripts of the movie from the Internet Movie Script Database^1^ and constructed a hypergraph for the eight main characters so that each character is a vertex in the hypergraph. We define each scene in the movie as a hyperedge with a total of 178 hyperedges, and a character is contained in the scene if he/she speaks in the scene.

We determine the optimal number of clusters and additional clusters using BIC where the results are provided in Table 6. The ELCA model with 3 clusters and 2 additional clusters has the lowest BIC value and is selected. It is worth noting that the standard LCA with 3 clusters is also competitive based on the BIC.

The results from fitting the ELCA model with $G=3$ and $K=2$ are provided in Tables 7 and 8. We can see the variation in the size of hyperedges from the parameter estimates ${\hat{a}}$ and ${\hat{\tau }}$ with the majority (81%) of hyperedges having size much smaller than the rest of the hyperedges. Thus, one can deduce that a small proportion of the movie scenes have far more characters.

**Table 6:** Model selection for the Star Wars data set

No. of clusters	No. of Additional clusters	BIC
1	1	1298.08
1	2	1437.86
2	1	1269.11
2	2	1271.55
3	1	1270.46
3	2	**1266.42**
3	3	1280.81
4	1	1273.54
4	2	1284.68
5	1	1307.05
5	2	1298.11
5	3	1306.50

The smallest value is bolded

**Table 7:** Estimates of $\pi$, $\tau$ and *a* from fitting the ELCA model with 3 clusters and 2 additional clusters for the Star Wars data set

${\hat{\pi }}$	(0.40, 0.40, 0.20)
$$ {\hat{\tau }} $$	(0.81, 0.19)
$$ {\hat{a}} $$	(0.41, 1.00)

**Table 8:** Estimates of $\{ \phi _{ig} \}$ from fitting the ELCA model with 3 clusters and 2 additional clusters for the Star Wars data set

Character	Cluster 1	Cluster 2	Cluster 3
Wedge	0.18	0.00	0.36
Han	0.00	1.00	0.00
Luke	1.00	1.00	0.00
C-3PO	0.75	0.30	0.00
Obi-Wan	0.00	0.00	1.00
Leia	0.12	0.48	0.07
Biggs	0.31	0.00	0.28
Darth Vader	0.19	0.35	0.06

The estimates ${\hat{\phi }}$ in Table 8 reveal interesting clustering structure for the 8 main characters in the movie. For example, the lead character “Luke” has a strong tendency to appear in the two largest clusters. On the other hand, it is extremely unlikely for “Obi-Wan” and “Han” appear in the same scene.

**Fig. 3:**
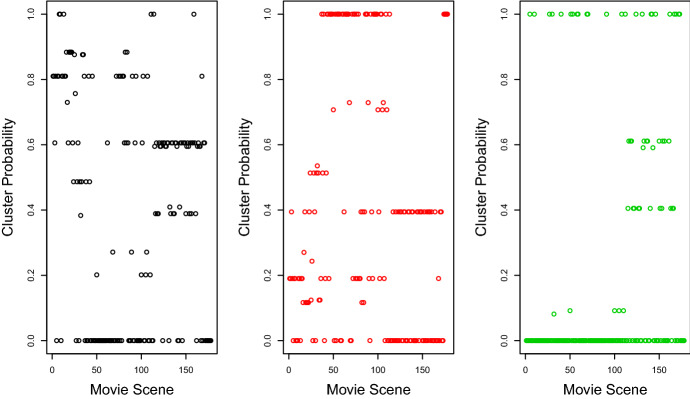
Probability of primary clusters for movie scenes in Star Wars data set plotted against movie scene number for the ELCA model with 3 primary clusters and 2 additional clusters. Cluster 1 is associated with scenes in the first half of the movie, whereas cluster 2 contains scenes mostly in the middle of the movie. On the other hand, scenes occuring in the second half of the movie are slightly more likely to be associated with cluster 3 compared to scenes ocurring in the first half of the movie

**Fig. 4:**
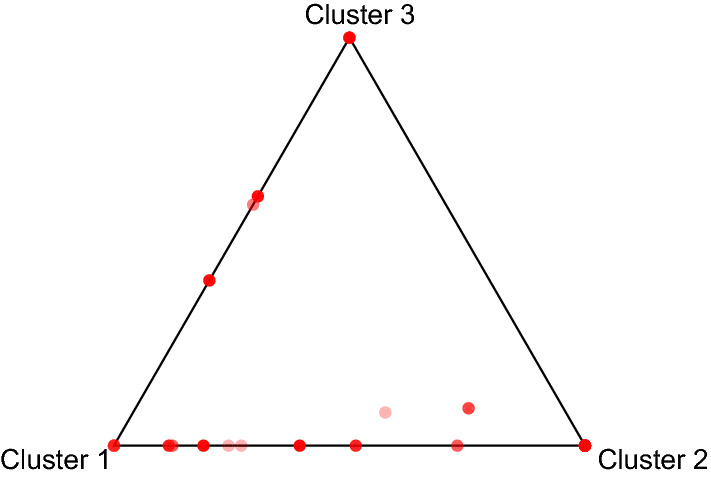
Ternary plot of the *a posteriori* group membership probabilities for the scenes in the Star Wars data set

The estimated primary cluster assignment probabilities from the EM algorithm for each movie scene in the Star Wars movie are shown in chronological order in Fig. 3. We can see from the plot that scenes in the early part of the movie are mainly associated with cluster 1, while cluster 2 contains most of the scenes from roughly scene 40 to scene 100. We can deduce from this, for example, that the character “Han” is very active in the middle part of the movie. On the other hand, there does not appear to be any obvious pattern for the third cluster. The clustering for many early and late movie scenes is relatively uncertain, as shown in the plot.

**Fig. 5:**
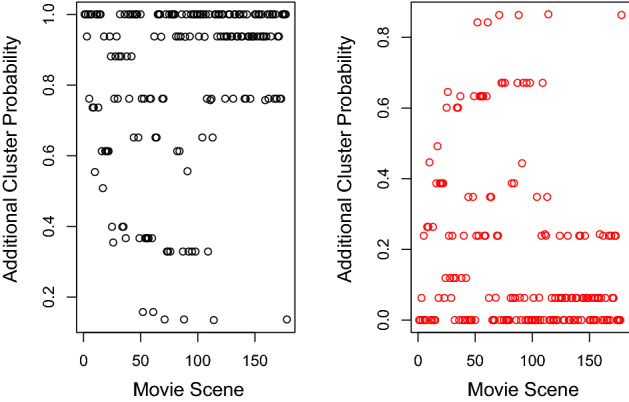
Probability of additional clusters for movie scenes in Star Wars data set plotted against movie scene number for the ELCA model with 3 primary clusters and 2 additional clusters. Majority of movie scenes are in cluster 1 whereas very few scenes are in cluster 2

The uncertainties in primary clustering are also illustrated in a ternary plot in Fig. 4. Each dot in the plot represents a movie scene, and the three corners of the plot represent the three clusters. The closer the dot is to the corner, the higher probability that the corresponding movie scene belongs to the corresponding cluster. The ternary plot in Fig. 4 shows significant uncertainties in clustering a number of movie scenes into the first two clusters. This is reasonable since for a number of actors including the lead actor “Luke”, the probabilities of scene appearance are similar for the first two clusters.

The estimated additional cluster assignment probabilities for each movie scene in the Star Wars movie are shown in chronological order in Fig. 5. We observe that majority of the scenes are assigned additional cluster 1 with only a small number of scenes between scene 40 and 100 assigned to additional cluster 2 where these scenes tend to have more characters.

**Fig. 6:**
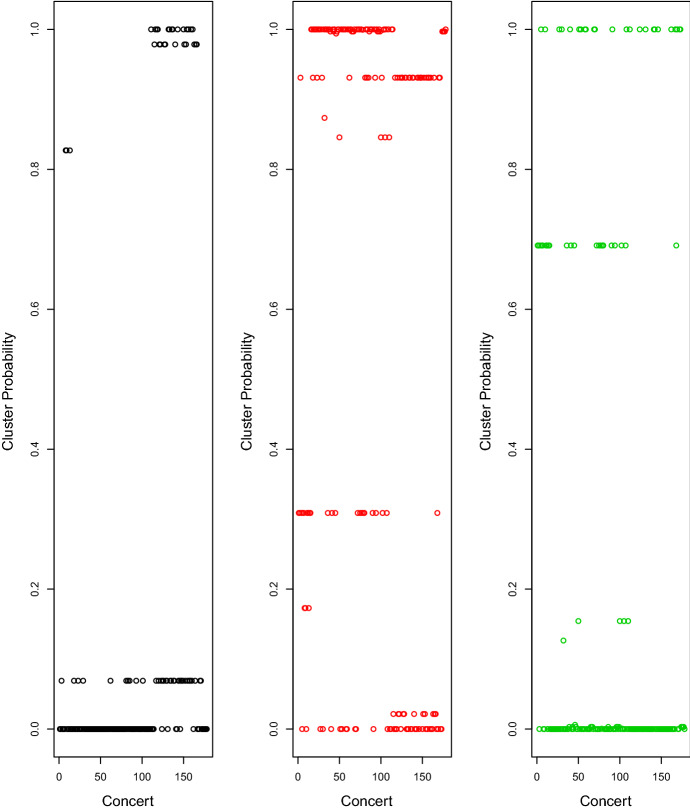
Probability of clusters for movie scenes in Star Wars data set plotted against movie scene number for the LCA model with 3 clusters. Movie scenes in cluster 1 mostly ocurred in the second half of the movie, whereas cluster 2 contains majority of the scenes in the movie. On the other hand, scenes in the first half of the movie are slightly more likely to be assoiated with cluster 3 compared to scenes in the second half of the movie

As a comparison, the results from fitting the standard LCA model with 3 clusters are shown in Tables 9 and 10, and a contigency table comparing the primary clustering structure of the ELCA model and the LCA model are given in Table 11. The contingency table shows a very different clustering structure obtained from fitting the standard LCA model versus the ELCA model. We show the estimated cluster assignment probabilities for each movie scene for the LCA model with 3 clusters in chronological order in Fig. 6. In comparing Fig. 3 with Fig. 6, we see that while primary cluster 2 and 3 for the fitted ELCA model are similar with cluster 2 and 3 for the fitted LCA model, there is significant difference between primary cluster 1 in the ELCA model and cluster 1 in the LCA model.

The difference in the clustering structure between the ELCA model and the LCA model is expected as the ELCA model explicitly captures the variation in the size of hyperedges. In comparison, the LCA model cannot decouple the variation in the size of hyperedges from the primary clustering structure. This is a key advantage of the ELCA model where the underlying structure of the size of the hyperedges can be uncovered. Furthermore, as a constrained version of the LCA model with 6 clusters, the ELCA model with 3 primary clusters and 2 additional clusters is far more parsimonious.

**Table 9:** Estimates of $\pi$ from fitting the LCA model with 3 clusters for the Star Wars data set

${\hat{\pi }}$	(0.17, 0.61, 0.22)

**Table 10:** Estimates of $\{ p_{ig} \}$ from fitting the LCA model with 3 clusters for the Star Wars data set

Character	Cluster 1	Cluster 2	Cluster 3
Wedge	0.47	0.00	0.00
Han	0.00	0.40	0.00
Luke	0.23	0.74	0.00
C-3PO	0.00	0.24	0.38
Obi-Wan	0.00	0.00	0.60
Leia	0.00	0.21	0.04
Biggs	0.52	0.02	0.00
Darth Vader	0.00	0.18	0.03

**Table 11:** Contingency table: ELCA with 3 clusters and 2 additional clusters versus LCA with 3 clusters

	LCA		
ELCA	1	2	3
1	16	47	22
2	0	57	0
3	12	0	24

### Reuters News articles

As a second application of the ELCA model, we collected news articles published by Reuters^2^ in January 2020. We analyze the co-appearance relationships among the Group of Eight+Five (G8+5) countries. A hypergraph is constructed by defining each news article as a hyperedge and each country as a vertex. A vertex is contained in a hyperedge if the corresponding country is mentioned in the corresponding news article. News articles that do not mention any of the 13 countries were removed, and the resulting hypergraph contains 1828 hyperedges.

**Table 12:** Model selection for Reuters News data set

No. of clusters	No. of additional clusters	BIC
1	1	18,018
1	2	19,005
2	1	17,801
2	2	17,711
2	3	17,723
3	1	17643
3	2	17636
3	3	17652
4	1	17562
4	2	17533
4	3	17625
5	1	17507
5	2	**17410**
5	3	17611
6	1	17468
6	2	17489
7	1	17514
7	2	17526

The smallest value is bolded

**Table 13:** Estimates of $\pi$, $\tau$ and *a* from fitting the ELCA model with 5 clusters and 2 additional clusters for Reuters News data set

${\hat{\pi }}$	(0.16, 0.27, 0.19, 0.12, 0.26)
$$ {\hat{\tau }} $$	(0.94, 0.06)
$$ {\hat{a}} $$	(0.28, 1.00)

**Table 14:** Estimates of $\{ \phi _{ig} \}$ from fitting the ELCA model with 5 clusters and 2 additional clusters for the Reuters News data set

Country	Cluster 1	Cluster 2	Cluster 3	Cluster 4	Cluster 5
BRA	0.19	0.27	0.00	0.42	0.00
CAN	0.00	0.27	**1.00**	**0.79**	0.00
CHN	**1.00**	**1.00**	0.46	0.62	0.79
DEU	0.00	0.49	0.38	0.19	**0.94**
FRA	0.00	**0.97**	0.80	0.00	**1.00**
GBR	0.39	0.79	**1.00**	0.32	**1.00**
IND	0.66	0.21	0.10	0.45	0.04
ITA	0.00	0.29	0.00	0.13	0.44
JPN	0.12	**1.00**	0.00	0.00	0.05
MEX	0.00	0.01	0.04	**0.95**	0.00
RUS	**0.95**	0.18	0.14	0.10	0.60
USA	**1.00**	0.35	**1.00**	**1.00**	0.47
ZAF	0.20	0.03	0.00	0.04	0.01

The largest three values in each column are bolded

The model with 5 clusters and 2 additional clusters was chosen by the BIC and fitted to the data set. The BIC scores for a range of models are shown in Table 12. It is worth noting that according to the BIC scores the ELCA models with two additional clusters generally outperform the standard LCA models whereas the standard LCA performs better than the ELCA with three additional clusters.

The parameter estimates ${\hat{\pi }}, {\hat{\tau }}$ and ${\hat{a}}$ are given in Table 13. The estimate ${\hat{\pi }}$ shows that the hyperedges are relatively evenly distributed across the five clusters. We can deduce from ${\hat{a}}$ and ${\hat{\tau }}$ that there are a small number of articles mentioning many countries whereas the vast majority of the articles mention very few countries. Specifically, about 6% of articles mentioned a much larger number of countries compared to the rest of the articles. The incorporation of an additional clustering structure results in significant reduction in the number of parameters.

The clustering structure can be deduced from the estimate ${\hat{\phi }}$ given in Table 14. China, Russia and USA are among the most popular in articles in cluster 1 whereas China, France and Japan are the most commonly mentioned by articles in cluster 2. Canada, Britain and USA have the highest probability of appearing in articles in cluster 3 whereas Canada, Mexico and USA are the most likely to appear in news articles in cluster 4. Germany, France and Britain are most likely to be mentioned by news articles in cluster 5 (Table 13).

## Conclusion

We have proposed the Extended Latent Class Analysis model as a generative model for random hypergraphs. Building on a proportionality assumption, the ELCA model introduces two clustering structures for hyperedges which captures variation in the size of hyperedges. The model achieves significant reduction in model complexity compared to the standard Latent Class Analysis model. An EM algorithm has been developed for model fitting where the M-step involves a series of conditional maximization and model selection is performed using BIC. The proposed model is fitted to two data sets and this yields interesting and interpretable structure within the vertices and hyperedges.

Several extensions to the ELCA model are possible. Hyperedges typically have temporal information associated with them, which is the case for the two applications in this paper. Developing a hypergraph model to incorporate such temporal information is of interest. Furthermore, while the ELCA is developed in the context of hypergraph applications, the model could be useful in other applications where the proportionality assumption on latent class conditional probabilities is plausible.

## Appendix A: Proof on Proposition 1

### Proof

We can write $A = \sum _{i=1}^{N} A_{i}$ where $A_{i}=1$ if node *i* appears in the hyperedge and $A_{i}=0$ otherwise. Similarly, we write $B=\sum _{i=1}^{N} B_{i}$. Let $Z_{A}$ be the latent cluster assignment of $X_{A}$ where $Z_{A} = g$ if $X_{A}$ is generated from cluster *g*. Let $Z_{B}^{(1)}$ and $Z_{B}^{(2)}$ be the latent cluster and additional clusters assignments of $X_{B}$, where $Z_{B}^{(1)}=g$ and $Z_{B}^{(2)}=k$ if $X_{B}$ is generated from cluster *g* and additional clusters *k*. We have

$$\begin{aligned} E(A)= & {} \sum _{g=1}^{G} E(A|Z_{A}=g) Pr(Z_{A}=g) \\= & {} \sum _{g=1}^{G} \sum _{i=1}^{N} E(A_{i} |Z_{A}=g) Pr(Z_{A}=g) \\= & {} \sum _{g=1}^{G} \sum _{i=1}^{N} p_{ig} \pi _{g} \\ E(B)= & {} \sum _{g=1}^{G} \sum _{k=1}^{K} E(B|Z_{B}^{(1)}=g, Z_{B}^{(2)}=k) Pr(Z_{B}^{(1)}=g, Z_{B}^{(2)}=k) \\= & {} \sum _{g=1}^{G} \sum _{k=1}^{K} \sum _{i=1}^{N} E(B_{i}|Z_{B}^{(1)}=g, Z_{B}^{(2)}=k) Pr(Z_{B}^{(1)}=g, Z_{B}^{(2)}=k) \\= & {} \sum _{g=1}^{G} \sum _{k=1}^{K} \sum _{i=1}^{N} \phi _{ig} a_{k} \tau _{k} \pi _{g} \\= & {} \sum _{g=1}^{G} \sum _{i=1}^{N} p_{ig} \pi _{g} \\= & {} E(A) \end{aligned}$$

For the variance of the LCA model, we have that

$$\begin{aligned} Var(A) = \sum _{i=1}^{N} Var( A_{i}) + 2 \sum _{i<j}^{N} Cov(A_{i}, A_{j}) \end{aligned}$$

where

$$\begin{aligned} Var(A_{i})= & {} E(A_{i}^{2}) - E(A_{i})^{2} \\= & {} Pr(A_{i}=1) - Pr(A_{i}=1)^{2} \\= & {} \sum _{g=1}^{G} p_{ig} \pi _{g} - \Big ( \sum _{g=1}^{G} p_{ig} \pi _{g} \Big )^{2} \\ \\ Cov(A_{i}, A_{j})= & {} E(A_{i} A_{j}) - E(A_{i}) E(A_{j}) \\= & {} Pr(A_{i}=A_{j}=1) - Pr(A_{i}=1) Pr(A_{j}=1) \\= & {} \sum _{g=1}^{G} p_{ig} p_{jg} \pi _{g} - \Big ( \sum _{g=1}^{G} p_{ig} \pi _{g} \Big ) \Big ( \sum _{g=1}^{G} p_{jg} \pi _{g} \Big ) \end{aligned}$$

Hence, we have that

$$\begin{aligned} Var(A)= & {} \sum _{i=1}^{N} \sum _{g=1}^{G} p_{ig} \pi _{g} - \sum _{i=1}^{N} \Big ( \sum _{g=1}^{G} p_{ig} \pi _{g} \Big )^{2} \\&+ 2 \sum _{i<j}^{N} \sum _{g=1}^{G} p_{ig} p_{jg} \pi _{g} - 2 \sum _{i<j}^{N} \Big ( \sum _{g=1}^{G} p_{ig} \pi _{g} \Big ) \Big ( \sum _{g=1}^{G} p_{jg} \pi _{g} \Big ) \end{aligned}$$

Now,

$$\begin{aligned} Var(B)= & {} \sum _{i=1}^{N} Var(B_{i}) + 2 \sum _{i<j}^{N} Cov(B_{i}, B_{j}) \\ Var(B_{i})= & {} Pr(B_{i}=1) - Pr(B_{i}=1)^{2} \\= & {} \sum _{g=1}^{G} \sum _{k=1}^{K} \phi _{ig} a_{k} \tau _{k} \pi _{g} - \Big ( \sum _{g=1}^{G} \sum _{k=1}^{K} \phi _{ig} a_{k} \tau _{k} \pi _{g} \Big )^{2} \\= & {} \sum _{g=1}^{G} p_{ig} \pi _{g} - \Big ( \sum _{g=1}^{G} p_{ig} \pi _{g} \Big )^{2} \\ Cov(B_{i}, B_{j})= & {} Pr(B_{i}=B_{j}=1) - Pr(B_{i}=1) Pr(B_{j}=1) \\= & {} \sum _{g=1}^{G} \sum _{k=1}^{K} \phi _{ig} \phi _{jg} a_{k}^{2} \pi _{g} \tau _{k} - \Big ( \sum _{g=1}^{G} p_{ig} \pi _{g} \Big ) \Big (\sum _{g=1}^{G} p_{jg} \pi _{g} \Big ) \\ \end{aligned}$$

We have

$$\begin{aligned} Var(B)= & {} \sum _{i=1}^{N} \sum _{g=1}^{G} p_{ig} \pi _{g} - \sum _{i=1}^{N} \Big ( \sum _{g=1}^{G} p_{ig} \pi _{g} \Big )^{2} \\&+ 2 \sum _{i<j}^{N} \sum _{g=1}^{G} \sum _{k=1}^{K} \phi _{ig} \phi _{jg} a_{k}^{2} \pi _{g} \tau _{k} - 2 \sum _{i<j}^{N} \Big ( \sum _{g=1}^{G} p_{ig} \pi _{g} \Big ) \Big ( \sum _{g=1}^{G} p_{jg} \pi _{g} \Big ) \end{aligned}$$

Now,

$$\begin{aligned} Var(B) - Var(A)= & {} 2 \sum _{i<j}^{N} \sum _{g=1}^{G} \sum _{k=1}^{K} \phi _{ig} \phi _{jg} a_{k}^{2} \pi _{g} \tau _{k} - 2 \sum _{i<j}^{N} \sum _{g=1}^{G} p_{ig} p_{jg} \pi _{g} \\= & {} 2 \sum _{i<j}^{N} \sum _{g=1}^{G} \Big ( \sum _{k=1}^{K} \phi _{ig} \phi _{jg} a_{k}^{2} \tau _{k} - p_{ig} p_{jg} \Big ) \pi _{g} \\= & {} 2 \sum _{i<j}^{N} \sum _{g=1}^{G} \phi _{ig} \phi _{jg} \Big ( \sum _{k=1}^{K} a_{k}^{2} \tau _{k} - \Big (\sum _{k=1}^{K} a_{k} \tau _{k} \Big )^{2} \Big ) \pi _{g} \end{aligned}$$

To show the quantity above is non-negative, we have to show that

$$\begin{aligned} \sum _{k=1}^{K} a_{k}^{2} \tau _{k} - \Big (\sum _{k=1}^{K} a_{k} \tau _{k} \Big )^{2} \ge 0 \end{aligned}$$

which follows from Jensen’s inequality. $\square$

## Appendix B: Simulation studies

Model parameters for Tables 1 and 3

$$\begin{aligned} a= & {} (0.5, 1) \\ \phi= & {} \begin{pmatrix} 0.8 &{} \cdots &{}0.8 &{} 0.1 &{} \cdots &{} 0.1 \\ 0.1 &{} \cdots &{} 0.1 &{} 0.8 &{} \cdots &{} 0.8 \\ \end{pmatrix} \end{aligned}$$

Model parameters for Tables 2 and 4

$$\begin{aligned} a= & {} (0.5, 1) \\ \phi= & {} \begin{pmatrix} 0.8 &{} \cdots &{}0.8 &{} 0.1 &{} \cdots &{} 0.1 \\ 0.1 &{} \cdots &{} 0.1 &{} 0.8 &{} \cdots &{}0.8 \\ 0.4 &{} \cdots &{} 0.4 &{} 0.4 &{} \cdots &{}0.4 \\ \end{pmatrix} \end{aligned}$$

## Footnotes

1 Movie script data freely available at https://www.imsdb.com/.

2 http://www.reuters.com.
